# Global Trends in Highly Cited Studies in COVID-19 Research

**DOI:** 10.1001/jamanetworkopen.2023.32802

**Published:** 2023-09-08

**Authors:** Satoshi Funada, Takashi Yoshioka, Yan Luo, Toshi Iwama, Chikako Mori, Naofumi Yamada, Hideki Yoshida, Kota Katanoda, Toshi A. Furukawa

**Affiliations:** 1 Department of Health Promotion and Human Behavior, School of Public Health, Graduate School of Medicine, Kyoto University, Kyoto, Japan; 2 Department of Preventive Medicine and Public Health, Keio University School of Medicine, Tokyo, Japan; 3 Office of Evidence and Analysis, Japan Society Technology and Agency, Tokyo, Japan; 4 Division of Surveillance and Policy Evaluation, National Cancer Center Institute for Cancer Control, Tokyo, Japan; 5 Population Health and Policy Research Unit, Medical Education Center, Graduate School of Medicine, Kyoto University, Kyoto, Japan

## Abstract

**Importance:**

Since the onset of the COVID-19 outbreak, an extremely high number of studies have been published worldwide, with variable quality. Research trends of highly cited papers may enable identification of influential research, providing insights for new research ideas; it is therefore important to investigate trends and focus on more influential publications in COVID-19–related studies.

**Objective:**

To examine research trends of highly cited studies by conducting a bibliometric analysis of highly cited studies in the previous 2 months about COVID-19.

**Design, Setting, and Participants:**

In this cross-sectional study, Essential Science Indicators (ESI) and Web of Science (WOS) Core Collection were used to find studies with a focus on COVID-19 that were identified as highly cited studies from Clarivate Analytics. Highly cited studies were extracted from the ESI database bimonthly between January 2020 and December 2022. Bibliographic details were extracted from WOS and combined with ESI data using unique accession numbers. The number of highly cited studies was counted based on the fractional counting method. Data were analyzed from January through July 2023.

**Main Outcomes and Measures:**

The number of publications by research field, country, and institutional affiliation.

**Results:**

The number of published COVID-19–related highly cited studies was 14 studies in January to February 2020, peaked at 1292 studies in November to December 2021, and showed a downward trend thereafter, reaching 649 studies in November to December 2022. China had the highest number of highly cited studies per 2-month period until July to August 2020 (138.3 studies vs 103.7 studies for the US, the second highest country), and the US had the greatest number of highly cited studies afterward (159.9 studies vs 157.6 studies for China in September to October 2020). Subsequently, the number of highly cited studies per 2-month period published by China declined (decreasing from 179.7 studies in November to December 2020 to 40.7 studies in September to October 2022), and the UK produced the second largest number of such studies in May to June 2021 (171.3 studies). Similarly, the top 5 institutional affiliations in May to June 2020 by highly cited studies per 2-month period were from China (Huazhong University: 14.7 studies; University of Hong Kong: 6.8 studies; Wuhan University: 4.8 studies; Zhejiang University: 4.5 studies; Fudan University: 4.5 studies), while in November to December 2022, the top 5 institutions were in the US and UK (Harvard University: 15.0 studies; University College London: 11.0 studies; University of Oxford: 10.2 studies; University of London: 9.9 studies; Imperial College London: 5.8 studies).

**Conclusions and Relevance:**

This study found that the total number of highly cited studies related to COVID-19 peaked at the end of 2021 and showed a downward trend until the end of 2022, while the origin of these studies shifted from China to the US and UK.

## Key Points

**Question:**

What is the global trend of highly cited studies investigating COVID-19 since its outbreak?

**Findings:**

This cross-sectional study found that the number of highly cited studies peaked at 1292 studies at the end of 2021 and declined to 649 studies at the end of 2022. Highly cited studies from China showed a decreasing trend, while those from the US and UK showed an increasing trend.

**Meaning:**

These findings suggest that as the COVID-19 pandemic evolved in the 3 years since its outbreak, there were important shifts in trends of the number and origin of high-profile COVID-19 studies.

## Teaser

This cross-sectional study investigates trends in publication of highly cited studies of COVID-19 worldwide from January 2020 to December 2022.

## Introduction

Since the outbreak of COVID-19 in December 2019, numerous studies have been conducted and published worldwide in response to the pandemic.^1^ This trend may have been amplified by the use of preprint systems, such as medRxiv^2^ and bioRxiv,^3^ during the pandemic, as well as by the proliferation of predatory journals.^4^ As a result, the total number of COVID-19–related publications, including preprints, has increased dramatically and now exceeds 350 000 studies.^5^ The dissemination of COVID-19 research is highly active and constantly evolving. In such an expanding research environment, investigating research trends may help identify knowledge gaps and provide insightful research directions.^6^ In addition, comparing trends across countries and institutional affiliations may support scientific policy and research management.^7^

However, as previously found in 2020,^8^ the increase in COVID-19–related publications has not necessarily been associated with increased high-quality evidence, and this concern has become a reality in 2023. A citation analysis of studies published in predatory journals found that 60% of publications had not attracted any citations and 38% were cited only up to 10 times.^9^ The COVID-19 pandemic has seemingly been associated with an exacerbated issue of waste of studies (ie, doing unnecessary or poorly designed studies),^10^ making the proper assessment and synthesis of research trends in COVID-19 research challenging. Therefore, some filtering system may be essential to efficiently narrow down desired publications from the vast collection and ensure that relevant and valuable studies are selected.

One way to address this challenge is to analyze highly cited studies, or hot papers, which refers to studies published within the previous 2 years that have received a considerable number of citations in the previous 2 months, placing them in the top 0.1% of studies in the same field.^11^ High citation counts indicate that these studies have garnered significant attention from researchers. Furthermore, the list is updated every 2 months, allowing researchers to keep up with the latest trends and analyze them over time to capture shifts in the research landscape. Examining research trends of highly cited studies may allow the identification of influential studies, providing valuable insights for generating new research ideas. Bibliometrics is a scientific domain focused on measuring and quantifying various features in publications by examining the productivity of researchers, affiliations, and countries in specific fields.^12^ Therefore, a bibliometric analysis may be appropriate for examining features of highly cited studies in COVID-19 research. To our knowledge, there have been no studies analyzing the trend of COVID-19–related highly cited studies.

This study aimed to investigate research trends of highly cited studies by conducting a bibliometric analysis of these studies on COVID-19 research. Additionally, by presenting these studies in chronological order, we aimed to identify changes in COVID-19 research trends.

## Methods

This cross-sectional study was a bibliometric analysis of highly cited studies and followed the Strengthening the Reporting of Observational Studies in Epidemiology (STROBE) reporting guideline. According to the Ethical Guidelines for Medical and Health Research Involving Human Subjects in Japan, institutional review board approval and participant consent were not required for this study because it used only published data.

### Eligibility Criteria

We included all studies with a focus on COVID-19 identified as highly cited studies from Clarivate Analytics. We excluded studies that contained keywords related to COVID-19 in the text but did not investigate COVID-19. There was no restriction on the type of studies included.

### Study Identification and Selection

This study used the following 5 selection steps for identifying highly cited studies on COVID-19. In step 1, we extracted a total of 18 periods of highly cited studies from the Essential Science Indicators (ESI) (Clarivate Analytics) database bimonthly from January 2020 to December 2022 (January to February 2020 through November to December 2020, January to February 2021 through November to December 2021, and January to February 2022 through November to December 2022). In step 2, based on a unique accession number associated with each highly cited study record in the ESI, we combined bibliographic details, such as abstract, document type, and others, from the Web of Science (WOS) Core Collection (Clarivate Analytics) with ESI data. The accession number is an identification number assigned to each study in WOS, and an individual study is identified by searching with the accession number in WOS. In step 3, we identified highly cited studies with a focus on COVID-19 from their titles and abstracts using the following search terms: “COVID-19” or “2019-nCoV” or “NOVEL 2019” or “CORONAVIRUS DISEASE 2019” or “SARS-COV-2” or “n-COV” or “COVID” or “CORONAVIRUS” or “SARS.” We excluded highly cited studies that did not contain predetermined keywords as non-COVID-19–related highly cited studies. In step 4, a total of 4 researchers (T.I., C.M., N.Y., and H.Y.) making pairs in rotation each independently reviewed titles, abstracts, and full texts and included COVID-19–related highly cited studies that fit eligibility criteria. Any disagreements or ambiguity between pairs were resolved through discussion or cross-check consultation with another researcher if required. In step 5, the same 4 researchers (T.I., C.M., N.Y., and H.Y.) checked how many duplicates were counted as highly cited studies between each period. We conducted this selection step once for each of 18 periods between January 2020 and December 2022.

### Data Items and Collection

We collected the following bibliographic information from the WOS database: titles, authors, corresponding authors, affiliations, publication journal, publication date, and research field. Based on this information, we used 3 variables to measure the trend of COVID-19–related research as follows: (1) The research fields variable included 22 ESI categories (agricultural science; biology and biochemistry; chemistry; clinical medicine; computer science; ecology/environment; economics and business; engineering; geosciences; immunology; materials science; mathematics; microbiology; molecular biology and genetics; multidisciplinary; neuroscience and behavior; pharmacology; physics; plant and animal science; psychiatry/psychology; social sciences, general; and space science).^13^ Each journal is assigned to 1 field, and the research published in that journal adopts that field assignment. (2) The countries variable included countries of affiliation of all co-authors for each study. (3) The affiliations variable included affiliations of all co-authors for each study.

### Statistical Analysis

The bibliometric analysis descriptively summarizes the number of COVID-19–related highly cited studies. We counted the number of highly cited studies based on the fractional counting method. Compared with the full counting method, which counts the full number of each co-author and institutional affiliation, the fractional counting method had a fractional weight of each co-author and institutional affiliation, and each publication had a total weight of 1.^14^ Highly cited study counts were compared between research fields, countries, and affiliations. As a sensitivity analysis, we performed the full counting method instead of the fractional counting method. We also performed the fractional counting method on countries and affiliations of corresponding authors as a sensitivity analysis. Data were analyzed using R statistical software version 4.3.1 (R Project for Statistical Computing). Data were analyzed from January through July 2023.

## Results

### Study Selection and Trend of Highly Cited Studies

**Figure 1.**
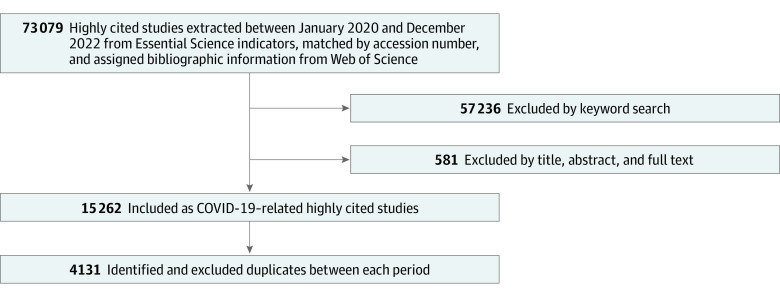
Study Flowchart

Figure 1 shows the selection step for highly cited studies on COVID-19 research. We identified 73 079 highly cited studies from the ESI database in 18 periods every 2 months between January 2020 and December 2022. From 73 079 highly cited studies, we excluded 57 236 highly cited studies by keyword search and 581 highly cited studies by title, abstract, and full text review. Finally, we identified 15 262 highly cited studies with duplicates and 4131 such studies without duplicates.

**Figure 2.**
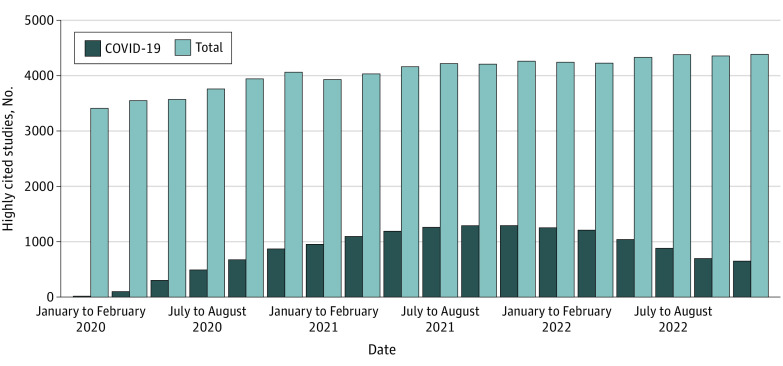
Trends of Research Fields Research fields of highly cited studies on COVID-19, including duplicates between each period, are presented.

Figure 2 shows the number of highly cited studies for COVID-19 research in each period. The total number of highly cited studies exhibited gradual growth, from 3412 studies in January to February 2020 to 4389 studies in November to December 2022. Regarding COVID-19–related highly cited studies, the initial count was 14 studies in January to February 2020, increasing to 1292 studies in November- to December 2021. However, there was a subsequent decline to 649 studies in November to December 2022.

The top 10 research fields of highly cited studies of COVID-19 in each period are given in eFigure 1 in Supplement 1. Although highly cited studies were predominantly from the clinical medicine field in January to February 2020 (9 of 14 studies [64.3%]), there was a gradual decrease in studies in this field starting in March to April 2022 (427 studies) until November to December 2022 (246 studies). Studies in other fields increased in number over time, with a particular increase in the fields of general social science, psychiatry and psychology, immunology, and molecular biology and genetics. For example, highly cited studies in general social science increased from 0 studies in January to February 2020 to 73 studies in July to August 2022.

### Comparison by Country

**Figure 3.**
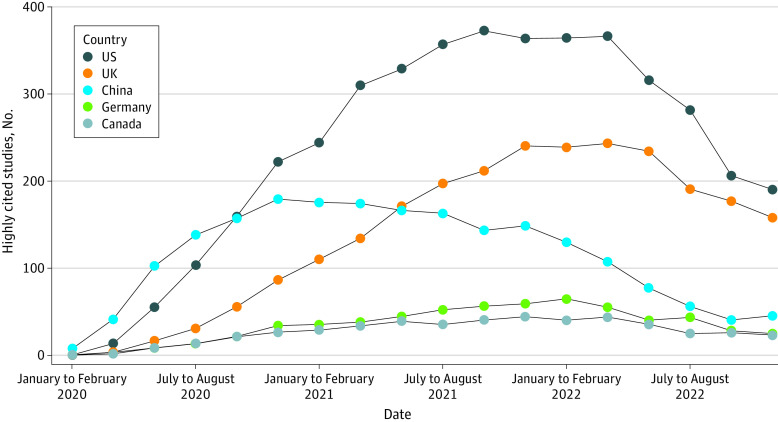
Top 5 Countries Producing Highly Cited Studies on COVID-19

Figure 3 shows the top 5 countries with the highest number of highly cited studies. China recorded the highest number of publications per 2-month period from January to February 2020 through July to August 2020 (138.3 studies), with the US following closely behind (103.7 studies during this period) and gradually increasing its output, overtaking China in September to October 2020 (159.9 studies vs 157.6 studies). China’s highly cited study output per 2-month period has been declining since November to December 2020 (decreasing from 179.7 studies in that period to 40.7 studies in September to October 2022), while there has been a steady increase in publications from the UK, increasing from 86.5 studies in November to December 2020 to ultimately overtake China in May to June 2021 (171.3 studies vs 166.6 studies). Starting in March to April 2022 until November to December 2022, the US, UK, and China had substantially reduced numbers of highly cited studies, and the downward trend continued until November to December 2022. The decrease in the number of highly cited studies per 2-month period from March to April 2022 to November to December 2022 was 366.8 studies to 190.6 studies for the US, 243.7 studies to 158.3 studies for the UK, and 107.5 studies to 45.5 studies for China. The trend remained the same using the full counting method (eFigure 2 in Supplement 1) and counting corresponding authors’ countries (eFigure 3 in Supplement 1) in sensitivity analyses.

### Comparison by Institutional Affiliation

**Figure 4.**
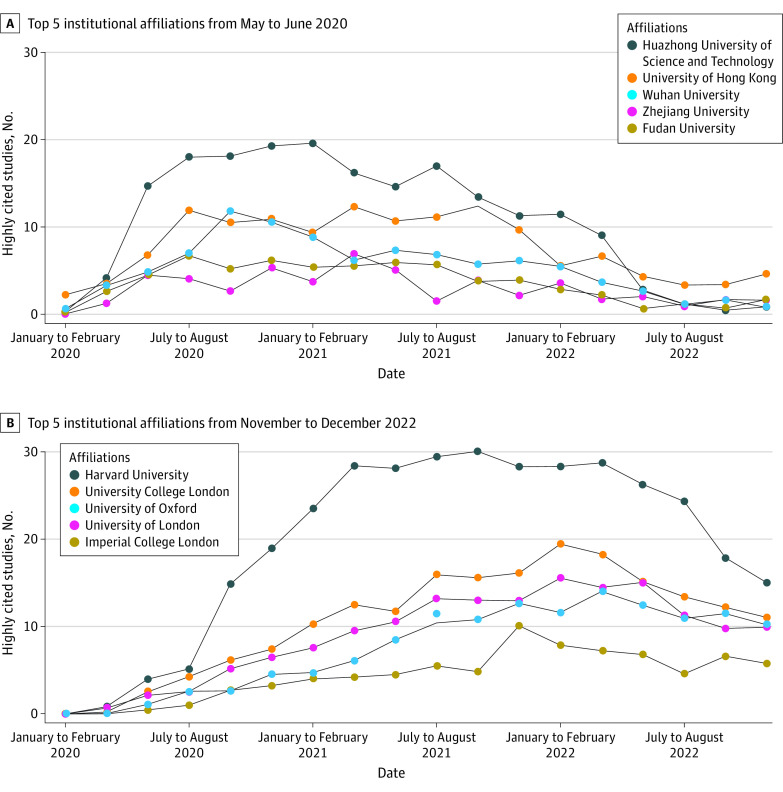
Top 5 Institutional Affiliations Producing Highly Cited Studies on COVID-19 Publication numbers are given over the entire study period for institutions with the most publications in A, May to June 2020 and B, November to December 2022.

Figure 4 shows the distribution of highly cited studies based on institutional affiliation across periods. Figure 4A and Figure 4B depict the top 5 facilities in terms of highly cited study publication numbers in May to June 2020 and November to December 2022, respectively. The top 5 institutional affiliations by highly cited studies per 2-month period in May to June 2020 were based in China (Huazhong University: 14.7 studies; University of Hong Kong: 6.8 studies; Wuhan University: 4.8 studies; Zhejiang University: 4.5 studies; Fudan University: 4.5 studies); however, by 2021, they all displayed a decreasing trend (Figure 4A). Conversely, in November to December 2022, the top 5 affiliations by highly cited studies per 2-month period were based in the US or the UK (Harvard University: 15.0 studies; University College London: 11.0 studies; University of Oxford: 10.2 studies; University of London: 9.9 studies; Imperial College London: 5.8 studies) (Figure 4B). Although there was some turnover, the trend remained the same in sensitivity analyses. There were more facilities in China in May to June 2020 by the full counting method (eFigure 4 in Supplement 1) and by affiliations of corresponding authors (eFigure 5 in Supplement 1) and more facilities in the US or UK in November to December 2022 by the full counting method (eFigure 4 in Supplement 1) and by affiliations of corresponding authors (eFigure 5 in Supplement 1).

**Figure 5.**
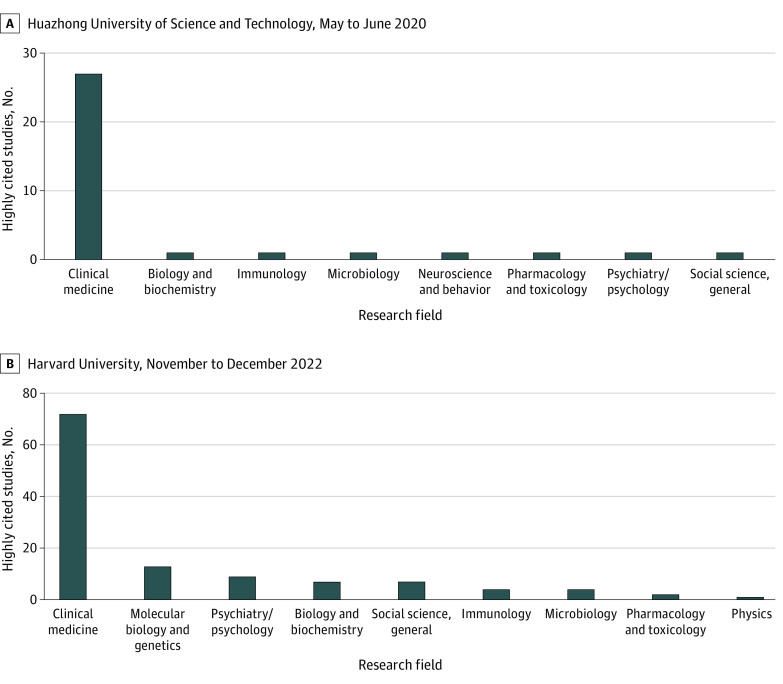
Research Fields of Highly Cited Studies From Top Affiliations Research fields are presented for the top affiliated institution in A, May to June 2020 (Huazhong University of Science and Technology) and B, November to December 2022 (Harvard University).

Figure 5 provides an overview of the research fields of affiliations with the highest number of highly cited studies in May to June 2020 (Figure 5A) and November to December 2022 (Figure 5B). Huazhong University of Science and Technology published the greatest number of highly cited studies in May to June 2020, with 27 of 34 studies in the clinical medicine field. In contrast, the top highly cited studies in November to December 2022 were from Harvard University, with 73, 13, and 9 highly cited studies in the fields of clinical medicine, molecular biology and genetics, and psychiatry and psychology, respectively.

## Discussion

### Principal Findings

This cross-sectional study evaluated trends in COVID-19 research by analyzing highly cited studies every 2 months from January 2020 to December 2022. As the pandemic progressed, the number of highly cited studies related to COVID-19 increased sharply. Nevertheless, after reaching a peak at the end of 2021, the number of highly cited studies exhibited a declining trend. In addition, while most highly cited studies were initially from the field of clinical medicine, we observed an increase in the number of publications from other fields through the observational period. Over time, there was a shift in the ranking of countries, with the US overtaking China to produce the highest number of highly cited studies since September to October 2020. The number of highly cited studies from China showed a decreasing trend, while those from the UK exhibited an increasing trend. Institutions that published the greatest number of highly cited studies at the beginning of the pandemic were from China; however, their number of publications gradually decreased, and the top institutions were replaced by those from the US and UK.

### Comparison With Other Studies

To our knowledge, no studies to date have conducted a bibliometric analysis of highly cited studies related to COVID-19 over the past 3 years. However, a bibliometric analysis using the COVID-19 Open Research Dataset (CORD-19)^15^ reported that COVID-19 studies, not just highly cited studies, published in 2020 came mostly from the US, China, and UK, which received more than 60% of citations. Similar to our research, that study found that the US steadily increased the number of studies and took the top spot, China had initially led COVID-19 research but experienced a substantial decline in research output over time, and the UK showed the opposite trend, starting with a slow pace of publications and gradually increasing its contributions throughout the year. Another study^16^ examined the association of COVID-19–related publications with overall publication rates in high-impact factor journals. They showed that studies related to COVID-19 accounted for approximately 10% to 50% of the total number of publications in each high-impact journal from 2020 to 2021. Additionally, a gradual decline in COVID-19–related publications was observed at the end of 2020. Notably, this declining trend was detected earlier in that study than in our analysis, suggesting a lag in citations given that highly cited studies are determined based on the number of citations after publication.

### Mechanisms and Implications

As reported in a previous study,^17^ an increase in COVID-19 cases in a region or country was associated with increased COVID-19–related research activity in that area. This may be associated with 2 factors: the need for data and increased government funding for research to control the pandemic. In addition, our findings of a gradual downward trend in highly cited studies related to COVID-19 may have been associated with a decrease in global attention to COVID-19 research. This trend may also suggest that researchers have gained a better understanding of the etiology and treatment of COVID-19, leading to decreased interest or fatigue with the topic.^18^ Changes in distribution, top affiliations, and research fields of highly cited studies suggest a gradual shift in interest in COVID-19 research toward more diversified and broader research areas. Based on results of this investigation, we expect a sustained reduction in the number of highly cited studies on COVID-19. Furthermore, we speculate that the research focus may further diversify, and we intend to examine this hypothesis through ongoing analysis. A bibliometric analysis using highly cited studies may be an appropriate method to capture trends in research by examining high-profile studies every 2 months. Similar methods used in this study may be useful for analyzing research trends in other fields.

### Strengths and Limitations

This study has several strengths. First, to our knowledge, it is the first bibliometric analysis of highly cited studies related to COVID-19. Investigations of highly cited studies (ie, those with the top 0.1% of citations) may be of greater interest than studies that analyze the total number of COVID-19–related studies. Using this method, we can exclude studies with low scientific impact, such as those published in predatory journals. In addition, highly cited studies are updated every 2 months, allowing the tracking of trends over time. Second, this study used fractional counting rather than full counting. While full counting is widely used in bibliometric analysis, fractional counting allows for field normalization and takes into account effects of aggregating large studies, particularly at the level of countries and research organizations. A comparative study^14^ recommended fractional counting in such bibliometric studies.

This study also has several limitations. First, while fractional counting is a strength, it can also be a limitation given that full counting is more widely used, making it challenging to compare our results with those of other studies. However, we also performed full counting as sensitivity analyses and observed no substantial difference in trends compared with fractional counting. This suggests that the difference in counting methods may not be a serious issue. Second, highly cited studies are limited to the top 0.1% of citations and are not representative of all published literature. In addition, the number of citations does not necessarily guarantee the quality of the research. Therefore, it should be noted that this study’s findings represent only trends in influential research. Third, although we used ESI categories to define research fields, these categories do not classify highly cited studies in detail. For example, clinical medicine includes a very broad range of highly cited studies. A more detailed classification may be appropriate for a closer look at research trends. Fourth, although this study identified trends in COVID-19–related highly cited studies, it provides a broad overview rather than a detailed analysis. Several interesting aspects could not be explored in depth in this study, including comparisons with non-COVID-19–related highly cited studies and the evolution of characteristic topics over time, such as lockdown policies and vaccines. To address these gaps, we need further analyses in the future.

## Conclusions

In this cross-sectional study, a bibliometric analysis of highly cited studies found that as the COVID-19 pandemic evolved over the 3 years since its outbreak, there was a shift in trends in COVID-19 research. The increase and decrease in the number of highly cited studies related to COVID-19 may suggest shifting interests of researchers. Meanwhile, there was a noticeable increase in the number of topics covered by field, including not only clinical medicine but also a diverse range of topics.

## Notes

Supplement 1. **eFigure 1.** Top Research Fields of Highly Cited Studies on COVID-19 **eFigure 2.** Top 5 Countries Producing Highly Cited Studies on COVID-19 Using Full Counting Method **eFigure 3.** Top 5 Countries of Corresponding Authors Producing Highly Cited Studies on COVID-19 **eFigure 4.** Top 5 Institutional Affiliations Producing Highly Cited Studies on COVID-19 Using Full Counting Method **eFigure 5.** Top 5 Institutional Affiliations of Corresponding Authors Producing Highly Cited Studies on COVID-19

Supplement 2.
Data Sharing Statement
