# Incorrect Institution Name in Author Affiliations

**DOI:** 10.1001/jamanetworkopen.2023.40819

**Published:** 2023-10-16

In the Original Investigation titled “Global Trends in Highly Cited Studies in COVID-19 Research,”^1^ published September 8, 2023, an error in the name of the institution Japan Science and Technology Agency in the author affiliations was fixed.^1^

## References

[1] Funada S, Yoshioka T, Luo Y, . Global trends in highly cited studies in COVID-19 research. JAMA Netw Open. 2023;6(9):e2332802. doi:10.1001/jamanetworkopen.2023.32802 37682572 PMC10492181

