# Supplementary material for: Global Trends in Highly Cited Studies in COVID-19 Research
Source: JAMA Netw Open. 2023 Sep 8;6(9):e2332802. doi: 10.1001/jamanetworkopen.2023.32802 (PMC10492181; doi:10.1001/jamanetworkopen.2023.32802)
Supplement: Supplement 1. — eFigure 1. Top Research Fields of Highly Cited Studies on COVID-19 eFigure 2. Top 5 Countries Producing Highly Cited Studies on COVID-19 Using Full Counting Method eFigure 3. Top 5 Countries of Corresponding Authors Producing Highly Cited Studies on COVID-19 eFigure 4. Top 5 Institutional Affiliations Producing Highly Cited Studies on COVID-19 Using Full Counting Method eFigure 5. Top 5 Institutional Affiliations of Corresponding Authors Producing Highly Cited Studies on COVID-19 [file jamanetwopen-e2332802-s001.pdf]

Supplemental Online Content

Funada S, Yoshioka T, Luo Y, et al. Global trends in highly cited studies in COVID-19 research. *JAMA Netw Open*. 2023;6(9):e2332802.
doi:10.1001/jamanetworkopen.2023.32802

eFigure 1. Top Research Fields of Highly Cited Studies on COVID-19

eFigure 2. Top 5 Countries Producing Highly Cited Studies on COVID-19 Using Full Counting Method

eFigure 3. Top 5 Countries of Corresponding Authors Producing Highly Cited Studies on COVID-19

eFigure 4. Top 5 Institutional Affiliations Producing Highly Cited Studies on COVID-19 Using Full Counting Method

eFigure 5. Top 5 Institutional Affiliations of Corresponding Authors Producing Highly Cited Studies on COVID-19

This supplemental material has been provided by the authors to give readers additional information about their work.

© 2023 Funada S et al. *JAMA Network Open.*

eFigure 1. (a) Top ten research fields of hot papers on COVID-19 research and **(b)** top nine research fields excluding Clinical Medicine

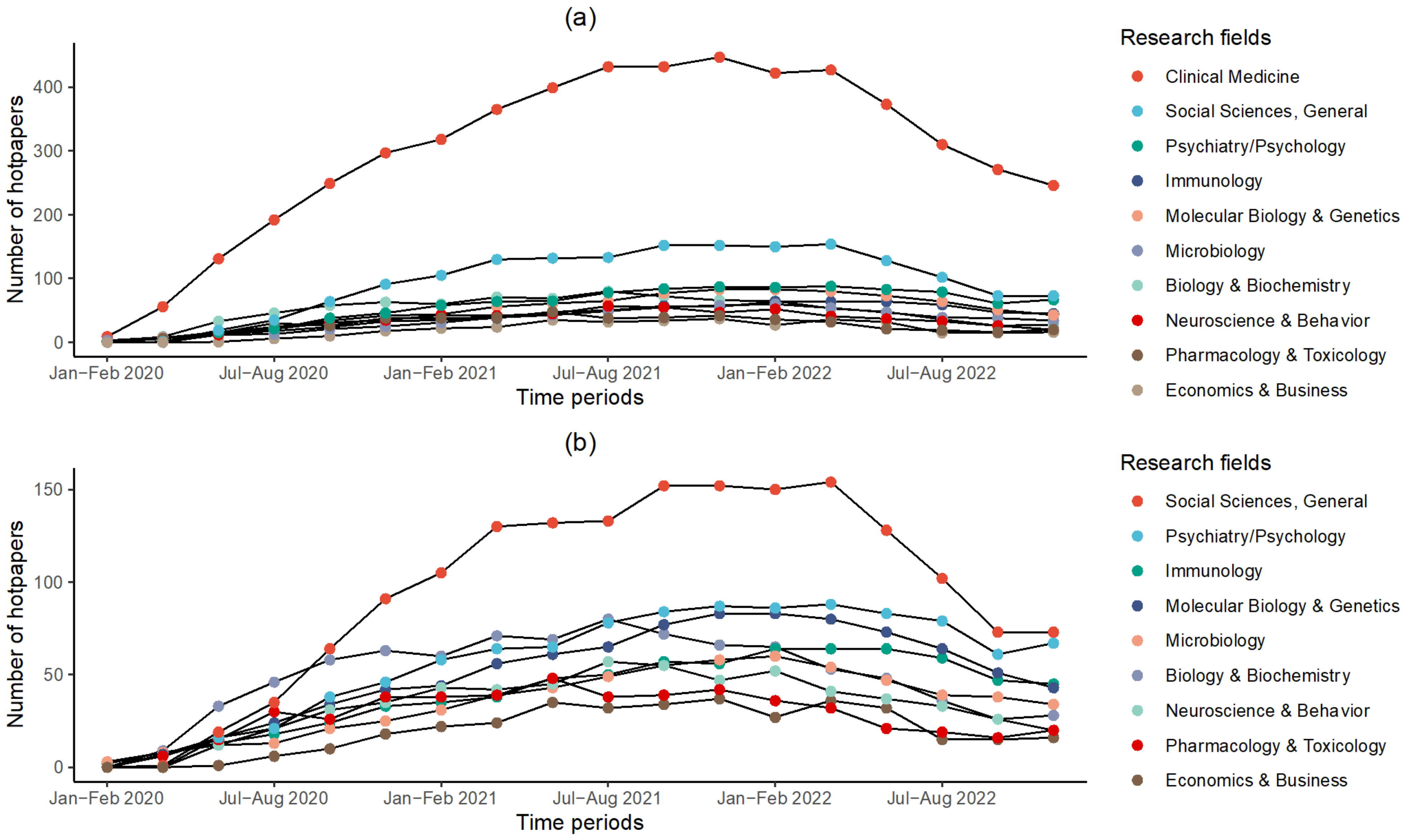

© 2023 Funada S et al. *JAMA Network Open.*

eFigure 2. Top five countries that produced hot papers on COVID-19 research using a full counting method (sensitivity analysis)

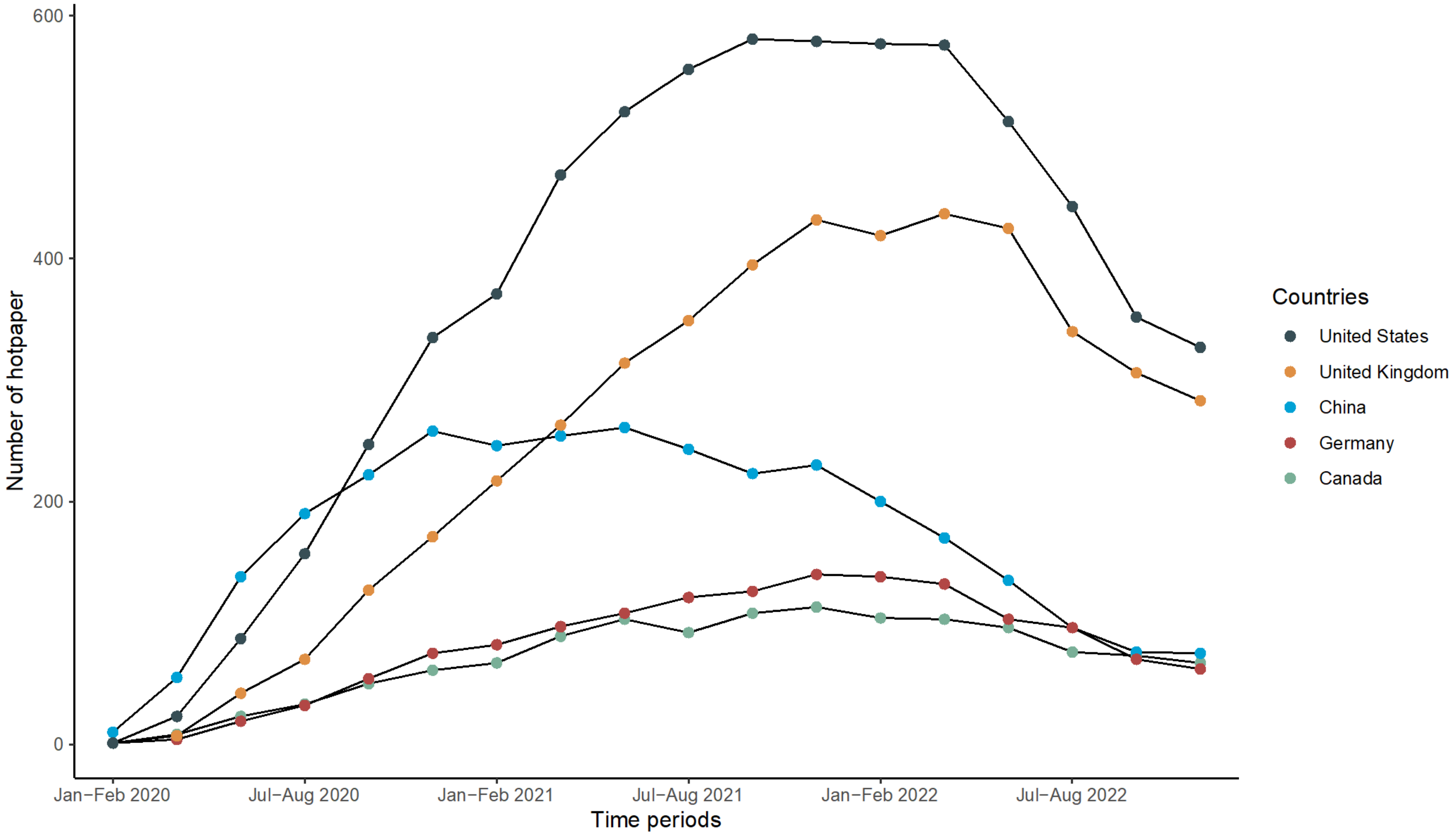

© 2023 Funada S et al. *JAMA Network Open.*

eFigure 3. Top five countries of corresponding authors that produced hot papers on COVID-19 research (sensitivity analysis)

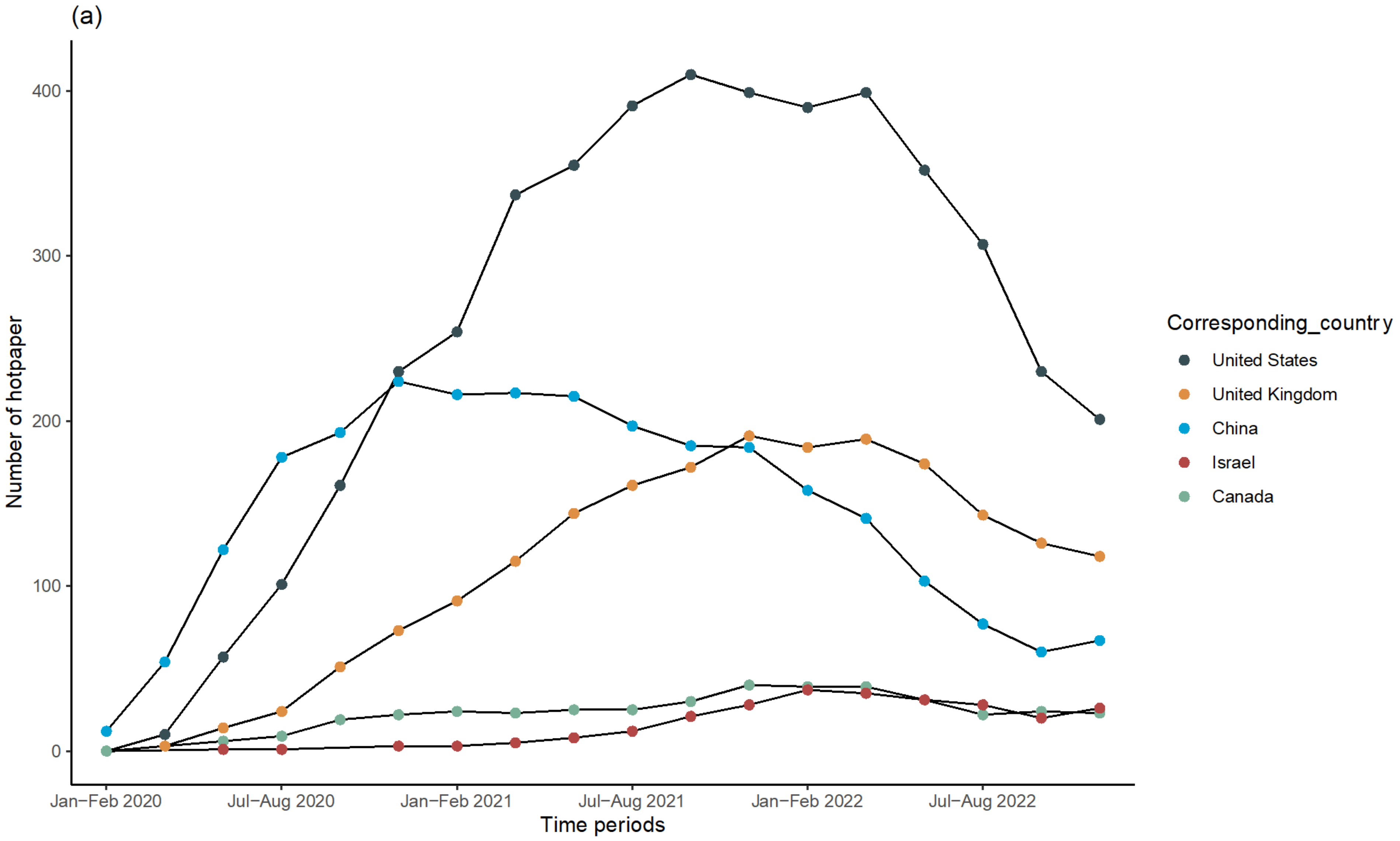

© 2023 Funada S et al. *JAMA Network Open.*

eFigure 4. Top five institutional affiliations that produced hot papers on COVID-19 research in **(a)** May-June 2020 and **(b)** November-December 2022 using a full counting method (sensitivity analysis)

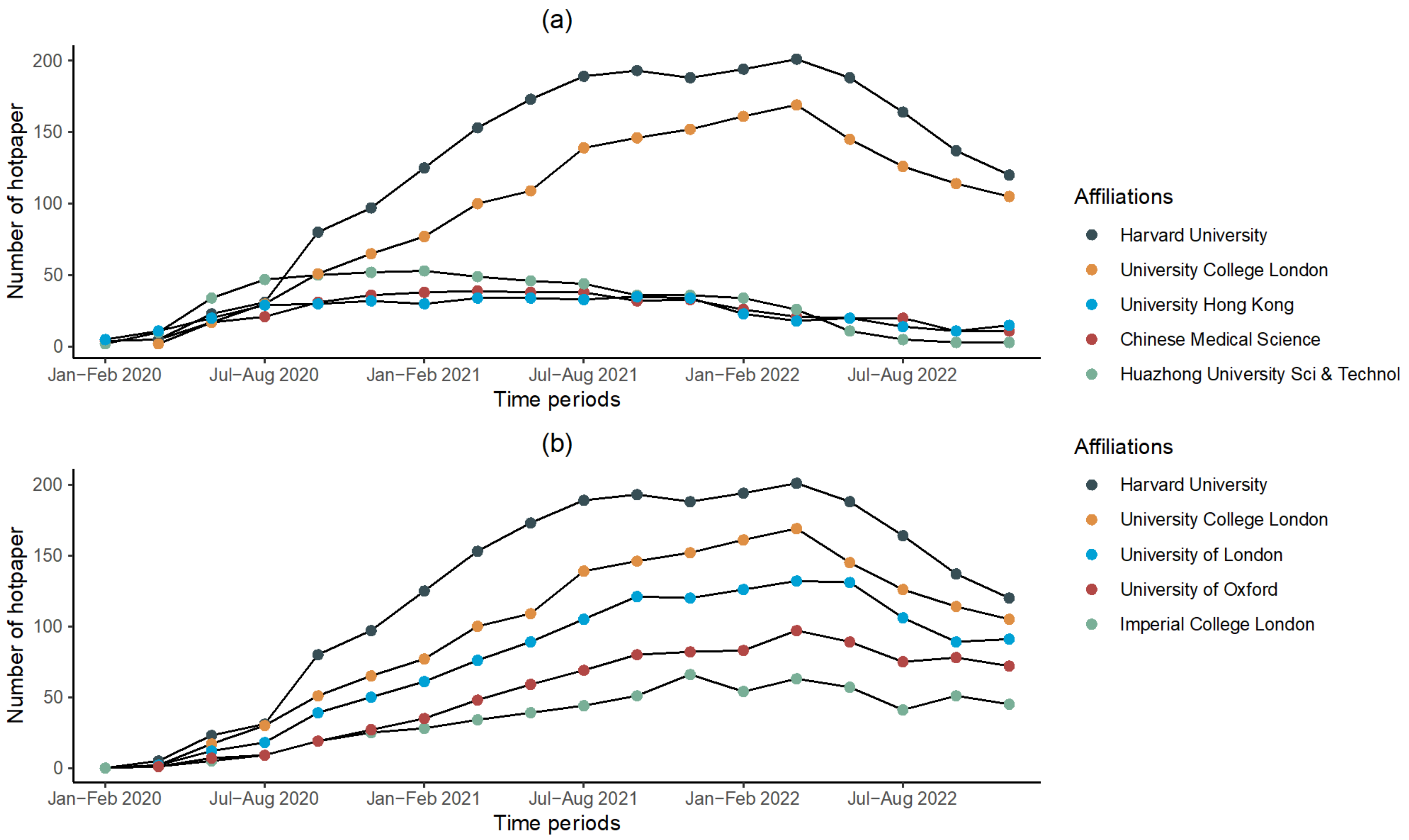

© 2023 Funada S et al. *JAMA Network Open.*

eFigure 5. Top five institutional affiliations of corresponding authors that produced hot papers on COVID-19 research in **(a)** May-June 2020 and **(b)** November-December 2022 (sensitivity analysis)

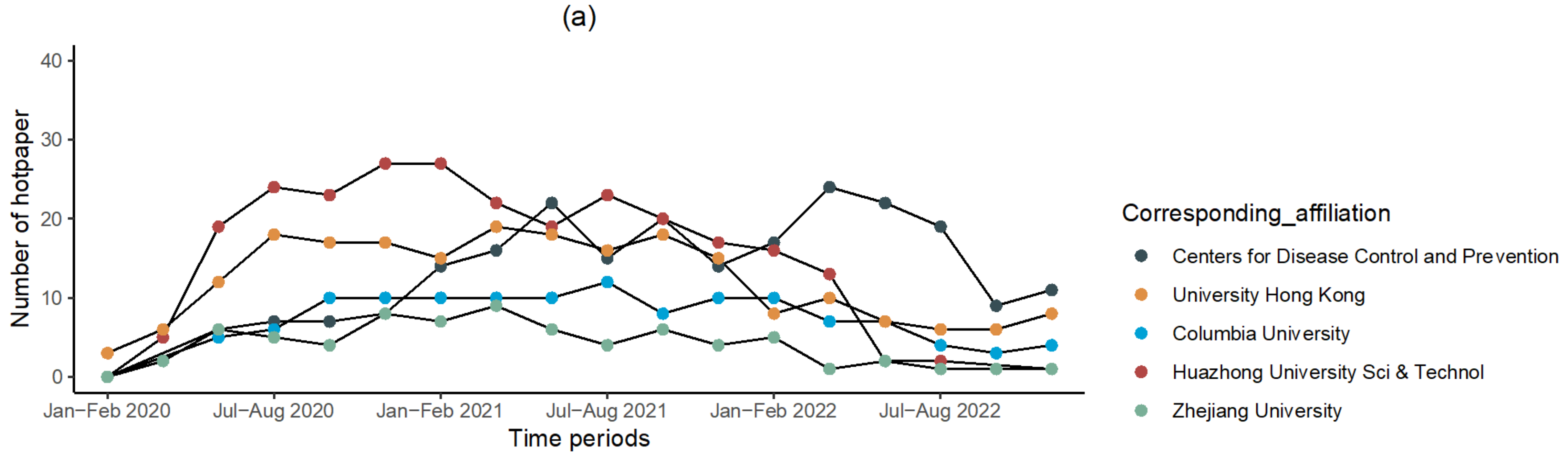

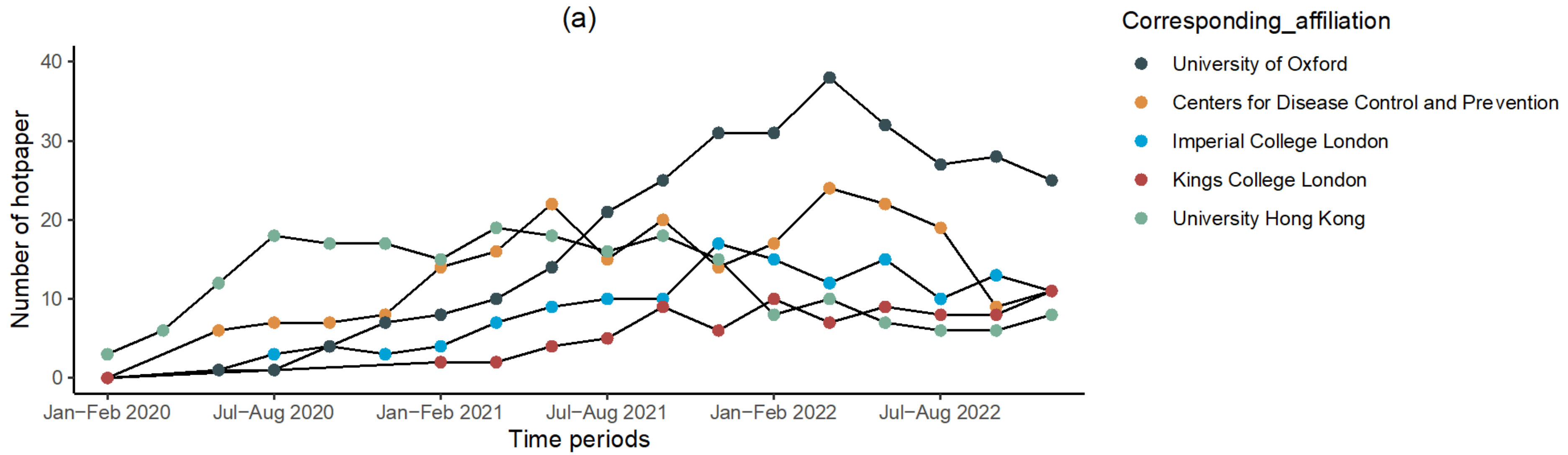

© 2023 Funada S et al. *JAMA Network Open.*
