# Supplementary material for: Global Trends in Highly Cited Studies in COVID-19 Research
Source: JAMA Netw Open. 2023 Sep 8;6(9):e2332802. doi: 10.1001/jamanetworkopen.2023.32802 (PMC10492181; doi:10.1001/jamanetworkopen.2023.32802)
Supplement: Supplement 2. — Data Sharing Statement [file jamanetwopen-e2332802-s002.pdf]

**Data Sharing Statement**

Funada. Global Trends in Highly Cited Studies in COVID-19 Research. *JAMA Netw Open*. Published online September 8, 2023. doi:10.1001/jamanetworkopen.2023.32802

**Data**
**Data available:** No
